# Micrometastases in sentinel nodes of gastric cancer

**DOI:** 10.1038/sj.bjc.6601183

**Published:** 2003-08-12

**Authors:** H Ajisaka, K Miwa

**Affiliations:** 1 Department of Gastroenterologic Surgery, Graduate School of Medical Science, Kanazawa University, Takara-machi 13-1, Kanazawa 920 8641, Japan

**Keywords:** sentinel node, micrometastasis, reverse transcription–polymerase chain reaction, southern blot assay, gastric cancer

## Abstract

The sentinel node (SN) is the first lymph node in the lymphatic basin to be affected by metastasis from the primary tumour and is used to predict the status of the remaining nodes in the basin. We succeeded in detecting SNs of clinically early gastric cancers by intraoperative injection of a blue dye around the tumour. In the study presented here, multiple-marker reverse transcription–polymerase chain reaction (RT–PCR) was used to detect micrometastases in SNs and results were compared with those obtained with conventional histology. Expressions of cytokeratin-18 (CK-18), carcinoembryonic antigen (CEA), human telomerase reverse transcriptase (hTRT) and MUC-1 in SNs were determined by RT–PCR and Southern blot assay. Of the 213 SNs obtained from 35 cases of gastric cancer, eight nodes (3.8%) from five patients contained metastases that could be identified by conventional histology. However, CK-18 mRNA was expressed in 15 (7.0%), CEA in 12 (5.6%), hTRT in 10 (4.7%), and MUC-1 in 12 (5.6%) nodes, with at least one mRNA marker expressed in 25 nodes (11.7%) obtained from six patients. In the five patients with nodal metastases identified by conventional histology, two had metastases in both SNs and non-SNs. And, in the 30 patients without nodal metastases identified by conventional histology, one patient with micrometastases in the SNs identified by RT – PCR and Southern blot assay also had metastases in non-SNs as identified by serial sectioning and immunostaining of CK-18. All additional metastases were detected in non-SNs located in the same lymphatic basin as the previously detected SNs. This suggests that lymph node dissection of early-stage gastric cancer in the lymphatic basin may be mandatory even for patients without histologically detectable metastases in SNs.

Lymph node metastasis is one of the most important determinants of whether minimally invasive surgery for early gastric cancer is appropriate, since lymph node dissection generally is considered unnecessary for early gastric cancer without nodal metastasis. Endoscopic mucosal resection or minimally invasive surgery without lymphadenectomy has recently been advocated for early gastric cancer without nodal metastasis. Predicting the absence of nodal metastasis is usually based on indirect evidence, by comparing the primary tumour in a particular case with those in other cases (Makuuchi *et al*, 1999). We succeeded in identifying sentinel nodes (SNs) in gastric cancer by intraoperative injection of blue dye around the tumour (Miwa *et al*, 2003). The SN is the first lymph node in the lymphatic basin to be affected by metastasis from the primary tumour and is used to predict the status of lymph nodes in the rest of the lymphatic basin (Morton *et al*, 1992). Metastases in the SNs are determined by intraoperative pathology, but if there is a discrepancy between the intraoperative diagnosis and postoperative diagnosis detailed examination results, a definitive operation cannot be performed only on the basis of a sentinel node biopsy (SNB). In this study, multiple-marker reverse transcription–polymerase chain reaction (RT–PCR) was used to detect micrometastases in SNs.

## MATERIALS AND METHODS

### Surgical specimens and blood preparation

Tissue specimens were obtained between April 1998 and May 1999 from 35 patients undergoing SNB just prior to gastrectomy for clinically early gastric cancer at the Department of Gastroenterologic Surgery, Graduate School of Medical Science, Kanazawa University. Of these patients, papillary adenocarcinoma, tubular adenocarcinoma, signet-ring cell carcinoma, and poorly differentiated adenocarcinoma were found in 1, 16, 8, and 10, respectively (Japanese Research Society for Gastric Cancer, 1995). Tumour location was upper third in seven, middle third in 19, and lower third in nine. SNB was performed in the lymphatic basins with stained lymphatic vessels and lymph nodes following injection of 0.2 ml patent blue at four points around the primary tumour by intraoperative endoscopy (Miwa *et al*, 2003). The type of gastrectomy was decided on location of the tumour and stained lymphatic basins. Consequently, wedge resection, segmental gastrectomy, proximal gastrectomy, distal gastrectomy, and total gastrectomy were performed for seven, eight, seven, nine, and four patients, respectively. Sentinel node biopsy showed 213 SNs. The nodes were bisected, and the halves were stored at −80°C until use. One-half of each node was examined by RT–PCR, and the other half by using conventional histology. Non-SNs dissected during gastrectomy following SNB generally were examined by conventional histology, and the serial sections were immunostained by using monoclonal antibodies specific to cytokeratin-18 (CK-18) (Dakopatts, Copenhagen, Denmark) to detect micrometastases. Lymphatic basins were classified into five directions: right and left gastric artery, right and left gastroepiploic artery, and posterior gastric artery (Coller *et al*, 1941). The relationship between the location of the gastric carcinoma and the number of SNs detected in each lymphatic basin is shown in Table 1

**Table 1:** Tumour location and the number of sentinel nodes (SNs) detected in each lymphatic basin

		**Lymphatic basin**				
**Location**	**No. of SNs (No. of patients)**	**L-GA**	**L-GEA**	**R-GA**	**R-GEA**	**P-GA**
Upper	34	26	6	0	0	2
	(7)	(6)	(2)	(0)	(0)	(1)
Middle	120	74	6	8	32	0
	(19)	(16)	(2)	(3)	(8)	(0)
Lower	59	9	0	2	48	0
	(9)	(4)	(0)	(1)	(9)	(0)

Values in parentheses are number of patients. L-GA=left gastric artery; L-GEA=left gastroepiploic artery; R-GA=right gastric artery; R-GEA=right gastroepiploic artery; P-GA=posterior gastric artery.

.

A total of 11 representative lymph nodes containing gastric cancer metastases and MKN-45, a gastric cancer cell line, served as positive controls and lymph nodes from five patients undergoing surgery for noncancerous conditions as negative controls. In addition, 10 ml of blood was collected in sodium citrate-containing tubes and centrifuged together with a hypotonic density-gradient solution, followed by collection of the nucleated cells in the blood. These peripheral blood lymphocytes from five healthy donors were then used as an additional negative control.

### RT–PCR and Southern blot assay

Total RNA was isolated by ISOGEN (Nippon Gene, Tokyo, Japan) according to the manufacturer's instructions. Reverse transcription was performed on the amount of total RNA specified for reverse transcriptase (Life Sciences, St Petersburg, FL, USA). The RNA was incubated at 68°C for 3 min with oligo (dT)_13_ primer and then put on ice before the RT reaction reagents were added. The RNA with RT reagents added was incubated at 37°C for 90 min, followed by heating at 95°C for 5 min. Polymerase chain reaction was performed using *Taq* DNA polymerase (Takara, Tokyo, Japan) and the identity of the PCR products was confirmed by Southern blot assay with a specific oligonucleotide probe. The target mRNAs were CK-18 (forward primer: TGGTCACCACACAGTCTGCT, reverse primer: CCAAGGCATCACCAAGATTA, probe: TGGAGGCCCGCTACGCCCTA), carcinoembryonic antigen (CEA) (forward primer: TCTGGAACTTCTCCTGGTCTCTCAGCTGG, reverse primer: TGTAGCTGTTGCAAATGCTTTAAGGAAGAAGC, probe: GGGCCACTGTCGGCATCATGATTGG), human telomerase reverse transcriptase (hTRT; forward primer: CGGAAGAGTGTCTGGAGCAA, reverse primer: CCCGTCACATCCACCTTGACAAAGTA, probe: GGATGAAGCGGAGTCTGGA), and MUC-1 (forward primer: CGTCGTGGACATTGATGGTACC, reverse primer: GGTA CCTCCTCTCACCTCCTCCAA, probe: TCCACCACCCTGTTGCTGTA). Glyceraldehyde-3-phosphate-dehydrogenase (G3PDH; forward primer: GCATCCTGGGCTACACTGAGC, reverse primer: GGTACATGACAAGGTGCGGC, probe: TCCACCACCCTGTTGCTGTA) was used as the endogenous control. The PCR conditions for CK-18, hTRT, and G3PDH were: 30 cycles at 95°C for 1 min, 50°C for 1 min, and 72°C for 1 min. The PCR conditions for MUC-1 were similar, except that only 20 cycles were performed. The PCR conditions for CEA were also similar, except that 46°C was used instead of 50°C. Reverse transcription–polymerase chain reaction conditions were controlled with a thermocycler (Astech, Fukuoka, Japan). Reverse transcription–polymerase chain reaction products were electrophoresed on a 2% agarose gel, transferred to a Hybond-N+ nylon membrane (Amersham, Buckinghamshire, England), and hybridised with an internal probe by an ECL 3′-oligolabelling detection system (Amersham). The washed membranes were autoradiographiccally exposed, followed by examination of hybridisation bands.

### Immunostaining

All specimens were fixed in 10% formalin, embedded in paraffin and cut into 4 *μ*m serial sections. The specimens were immunostained for CK-18 with the avidin–biotin–peroxidase complex method. After deparaffinisation, the sections were treated with 0.3% hydrogen peroxide in methanol for 30 min to block endogenous peroxidase and washed with phosphate-buffered saline. The specimens were then incubated with normal goat serum for 30 min to eliminate nonspecific staining and reacted overnight at 4°C with the anti-CK-18 monoclonal antibody diluted to 1 : 100. Peroxidase-labelled goat anti-rabbit and anti-mouse immunoglobulin (envision labelled polymer reagent) (Dakopatts) were applied for 60 min as secondary antibodies. Finally, diaminobenzidine was added for colour development and methyl green was used for nuclear staining. Normal rabbit IgG was used instead of the primary antibody for the negative control.

### Micrometastasis

In this study, micrometastasis was defined as the metastasis that was detected only by RT–PCR and Southern blot assay or immunostaining.

## RESULTS

### Tumour marker mRNA expression in controls

**Figure 1:**
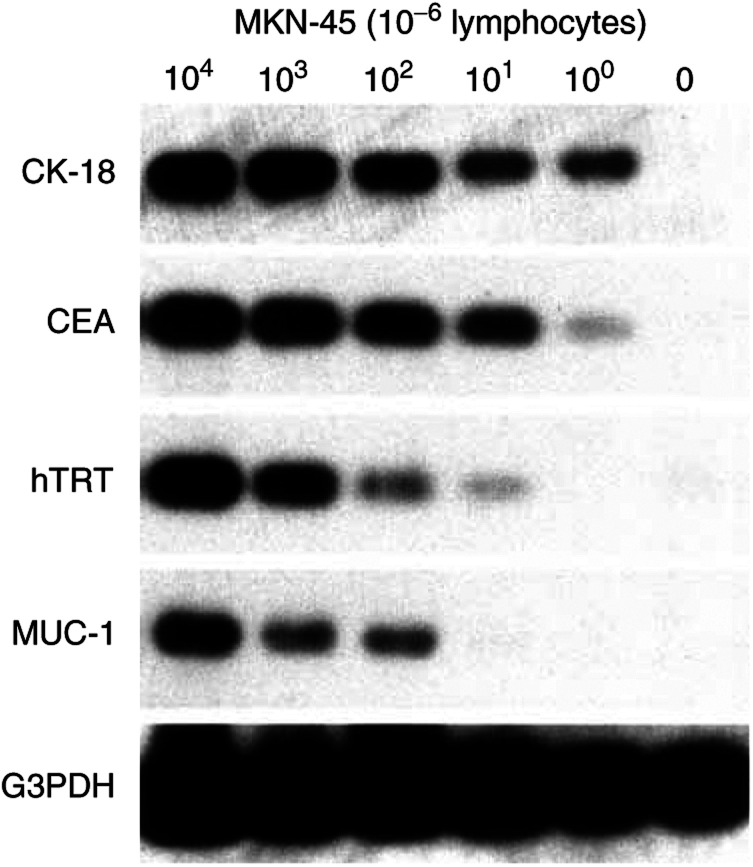
Sensitivities of RT–PCR and Southern blot assay of mRNA markers for gastric cancer. In 10^6^ lymphocytes, one cell of the MKN-45 gastric cancer cell line could be detected by CK-18 and CEA, 10 cells could be detected by hTRT, and 100 cells could be detected by MUC-1.

None of the four mRNA markers, CK-18, CEA, hTRT, or MUC-1, was expressed in any of the negative controls, that is lymphocytes from five healthy donors and five lymph nodes obtained from patients without cancer. CK-18 was expressed in eight (73%) of 11 lymph nodes containing gastric cancer metastasis, CEA in seven (64%), hTRT in six (55%), and MUC-1 in six (55%). In other words, at least one mRNA marker was expressed in all positive controls. CK-18 and CEA could detect 1 MKN-45 cell in 10^6^ lymphocytes, hTRT 10 cells, and MUC-1 100 cells (Figure 1

).

### Tumour marker mRNA expression in SNs

**Figure 2:**
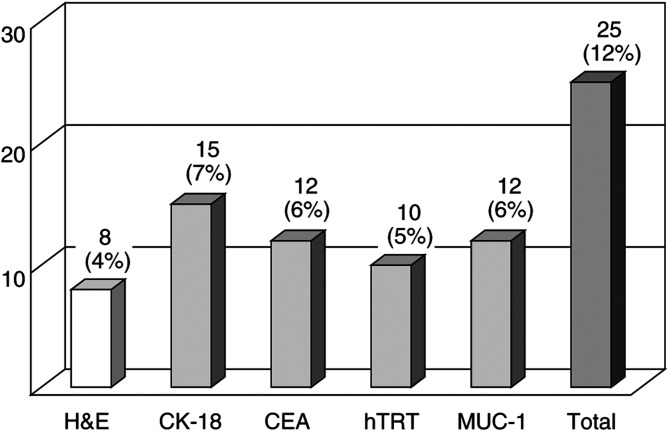
Number of metastases detected by conventional histology, haematoxylin and eosin (H&E) staining, and RT–PCR and Southern blot assay in 213 sentinel nodes. CK-18=cytokeratin-18; CEA= carcinoembryonic antigen; hTRT=human telomerase reverse transcriptase.

Of the 213 SNs obtained from 35 patients, eight (4%) from five patients contained metastasis detected by conventional histology. All of metastatic deposits exhibited the same histological type of the primaries. CK-18 mRNA was expressed in 15 SNs (7%), CEA in 12 (6%), hTRT in 10 (5%), and MUC-1 in 12 (6%). At least one mRNA marker was expressed in 25 nodes (12%) harvested from six patients (Figure 2

). All eight SNs with metastasis detected by conventional histology expressed at least one mRNA marker in RT–PCR and Southern blot assay, while 17 (8%) of 205 SNs without histologically detectable metastasis expressed at least one mRNA marker (Table 2

**Table 2:** Comparison of metastases in sentinel nodes (SNs) detected by conventional histology and by reverse transcriptase–polymerase chain reaction (RT–PCR) and Southern blot assay in the six patients with nodal metastases

		**Metastasis**				
**Patient no. (location/histological type)**	**Lymphatic basin**	**H&E**	**CK-18**	**CEA**	**hTRT**	**MUC-1**
1	L-GA	+	−	−	−	+
(Upper/Por)	L-GA	−	−	+	−	+
	L-GA	−	−	+	−	−
						
2						
(Lower/Pap)	R-GEA	+	−	−	+	+
	R-GEA	−	−	−	+	−
	R-GEA	−	−	−	+	+
						
3	R-GEA	+	+	+	−	+
(Lower/Sig)	R-GEA	+	+	+	−	−
	R-GEA	+	+	−	−	+
	R-GEA	−	+	+	−	+
	R-GEA	−	+	+	−	+
	R-GEA	−	−	+	−	−
						
4	L-GEA	+	+	+	−	−
(Upper/Tub)	L-GEA	−	+	+	+	−
						
5						
(Lower/Por)	R-GEA	+	+	+	+	−
	R-GEA	+	+	+	−	−
	R-GEA	−	+	−	+	+
	R-GEA	−	+	−	+	−
	R-GEA	−	+	−	−	+
	R-GEA	−	+	−	−	−
	R-GEA	−	−	+	+	−
	R-GEA	−	−	−	+	+
	R-GEA	−	−	−	+	−
						
6	L-GA	−	+	−	−	−
(Upper/Tub)	L-GA	−	+	−	−	−

Nos. 4 and 5 had metastases in non-SNs detected by conventional histology. No. 6 had metastases in non-SNs detected by immunostaining for CK-18. Pap=papillary adenocarcinoma; Tub=tubular adenocarcinoma; Sig=signet-ring cell carcinoma; por=poorly differentiated adenocarcinoma; L-GA=left gastic artery; L-GEA=left gastroepiploic artery; R-GEA=right gastroepiploic artery; CK-18=cytokeratin-18; CEA=carcinoembryonic antigen; hTRT=human telomerase reverse transcriptase.

).

Two of the five patients with metastases in SNs detected with conventional histology also showed metastases in non-SNs (Table 3

**Table 3:** Comparison of metastases in sentinel nodes (SNs) and in non-SNs detected by conventional histology and by reverse transcriptase–polymerase chain reaction (RT–PCR) and Southern blot assay

**Conventional histology**			** RT–PCR and Southern blot assay**		
**SN metastasis**	**Non-SN metastasis**		**SN metastasis**	**Non-SN metastasis**	
	**Negative**	**Positive**		**Negative**	**Positive**
Negative	30	0	Negative	29	0
Positive	3	2^a^	Positive	3	3^a^

a All of the metastatic non-SNs were located in the same lymphatic basin as the metastatic SNs.

). And, of the 30 patients without metastases in SNs detected with conventional histology, one patient with metastases in SNs detected by RT–PCR and Southern blot assay also showed metastases in non-SNs when immunostaining for CK-18 was used. All of the metastatic non-SNs of these three patients were identified in the same lymphatic basin as the metastatic SNs.

## DISCUSSION

We have been using blue dye injection to identify SNs in gastric cancer since 1993 (Miwa *et al*, 2003). If SNs do not contain metastases, minimally invasive surgery without extended dissection is possible. However, this method of determining the indication for minimally invasive surgery has several shortcomings. The first is that some micrometastases cannot be detected by intraoperative pathology or conventional histology. The second problem is how to determine the appropriate extent of lymph node dissection in cases where SNs do contain metastases.

Several investigators have reported using ultrasensitive molecular techniques for RT–PCR detection of micrometastasis in blood, bone marrow, and lymph nodes (Rosen *et al*, 1981; Ioguchi *et al*, 1994; Brown *et al*, 1995; Mori *et al*, 1995; Bostick *et al*, 1998; Takakura *et al*, 1998). This method has increased the detection rate for occult metastases as a result of amplification of mRNA tumour markers or epithelial markers and it has been suggested that patients exhibiting a positive band for these mRNAs in lymph nodes had a significantly worse prognosis than those who were RT–PCR-negative (Natsugoe *et al*, 1998; Cai *et al*, 1999; Noura *et al*, 2002). Although tumours are heterogeneous in gene expression, most studies have relied on a single marker to detect micrometastasis, and it has been difficult to improve not only the sensitivity but also the specificity of the RT–PCR detection assays. The use of multiple markers is one strategy for improving both sensitivity and specificity (Bostick *et al*, 1998). We assessed the presence of micrometastases in SNs of gastric cancer by using multiple-marker RT–PCR and Southern blot assay with the markers CK-18 (Brown *et al*, 1995), CEA (Mori *et al*, 1995), hTRT (Takakura *et al*, 1998), and MUC-1 (Noguchi *et al*, 1994).

In addition to eight metastases detected in 213 SNs by conventional histology, 17 additional micrometastases were detectable by RT–PCR and Southern blot assay. Of these 17 SNs, 15 were seen in five patients with histological nodal metastases and were present in the same lymphatic basin as the metastatic SNs detected with conventional histology. The other two SNs were detected in one patient diagnosed as negative for nodal metastasis by conventional histology.

The lymphatic basins with lymphatic vessels and lymph nodes stained with our blue dye injection technique can also be classified into five areas: right and left gastric artery, right and left gastroepiploic artery, and posterior gastric artery (Coller *et al*, 1941). In this study, non-SNs were evaluated by serial sectioning and immunostaining in addition to conventional histology, and metastatic non-SNs were detected in two patients with metastatic SNs detected by conventional histology and in one patient with micrometastatic SNs seen on RT–PCR and Southern blot assay. All of these metastatic non-SNs were located in the same lymphatic basin as the metastatic SNs.

As the result of detailed examination using RT–PCR and Southern blot assay or serial sectioning and immunostaining, all additional lymph node metastases were detected in non-SNs located in the same lymphatic basin as the previously detected metastatic SNs. This means that dissection of the lymphatic basin containing SNs is minimal requirement for curative resection of early-stage gastric cancer, even for patients without histologically detectable metastases in SNs.
